# 

**Published:** 1903-12

**Authors:** 


SUBSCRIPTION $1.00.

PUBLISHED MONTHLY

### ATLANTA JOURNAL-RECORD OF MEDICINE.

Successor to Atlanta Medical and Surgical Journal, Established 1855, and Southern Medical Record, Established 1870.

VOL. V. ATLANTA, GA., DECEMBER, 1903. NO. 9.

